# Evaluation of Threshold Values for Root Canal Filling Voids in Micro-CT and Nano-CT Images

**DOI:** 10.1155/2018/9437569

**Published:** 2018-07-16

**Authors:** Kaan Orhan, Reinhilde Jacobs, Berkan Celikten, Yan Huang, Karla de Faria Vasconcelos, Laura Ferreira Pinheiro Nicolielo, Arda Buyuksungur, Jeroen Van Dessel

**Affiliations:** ^1^Faculty of Dentistry, Department of Dentomaxillofacial Radiology, Ankara University, Ankara, Turkey; ^2^OMFS IMPATH Research Group, Department of Imaging & Pathology, Faculty of Medicine, University of Leuven and Oral & Maxillofacial Surgery, University Hospitals Leuven, Leuven, Belgium; ^3^Oral Facial Diagnostics and Surgery, Department of Dental Medicine, Karolinska Institutet, Solna, Sweden; ^4^Faculty of Dentistry, Department of Endodontics, Ankara University, Ankara, Turkey; ^5^State Key Laboratory of Oral Diseases, West China College of Stomatology, Sichuan University, Chengdu, China; ^6^Department of Oral Diagnosis, Division of Oral Radiology, Piracicaba Dental School, State University of Campinas, Piracicaba, Brazil; ^7^BIOMATEN, Middle East Technical University, Ankara, Turkey

## Abstract

While several materials and techniques have been used to assess the quality of root canal fillings in micro-CT images, the lack of standardization in scanning protocols has produced conflicting results. Hence, the aim of this study was to determine a cutoff voxel size value for the assessment of root canal filling voids in micro-CT and nano-CT images. Twenty freshly extracted mandibular central incisors were used. Root canals were prepared with nickel titanium files to an ISO size 40/0.06 taper and then filled with a single cone (40/0.06 taper) and AH Plus sealer. The teeth were scanned with different voxel sizes with either micro-CT (5.2, 8.1, 11.2, and 16.73 *μ*m) or nano-CT (1.5 and 5.0 *μ*m) equipment. Images were reconstructed and analyzed with the NRecon and CTAn software. Void proportion and void volume were calculated for each tooth in the apical, middle, and coronal thirds of the root canal. Kruskal-Wallis and post hoc Mann–Whitney *U* tests were performed with a significance level of 5%. In micro-CT images, significantly different results were detected among the tested voxel sizes for void proportion and void volume, whereas no such differences were found in nano-CT images (*p* > 0.05). Micro-CT images showed higher void numbers over the entire root length, with statistically significant differences between the voxel size of 16.73 *μ*m and the other sizes (*p* < 0.05). The values of the different nano-CT voxel sizes did not significantly differ from those of the micro-CT (5.2, 8.1, and 11.2 *μ*m), except for the voxel size of 16.73 *μ*m (*p* < 0.05). All tested voxel sizes enabled the detection of root canal filling voids except for the voxel size of 16.73 *μ*m. Bearing in mind the limitations of this study, it seems that a voxel size of 11.2 *μ*m can be used as a reliable cutoff value for the assessment of root canal filling voids in micro-CT imaging.

## 1. Introduction

The purpose of filling a root canal after endodontic instrumentation is to prevent reinfection [1]. Various materials and techniques have been investigated regarding the quality of root canal fillings using different assessment methods [2–5], and the development of novel imaging technologies has the potential of providing new insights on that particular issue. Microcomputer tomography (micro-CT), for instance, has contributed to the development of new assessment methodologies concerning the quality of root canal fillings [6]. Micro-CT is similar to CT except that the reconstructed, nano-sized cross sections are focused on a much smaller region of interest. It is a noninvasive, nondestructive technique commonly used for the analysis of mineralized tissues derived from the geometrical concept of cone-beam CT. In fact, cone-beam micro-CT combines a microfocus X-ray source with a high-resolution detector that can achieve spatial resolutions down to a few micrometers [7]. The image quality of devices using the cone-beam geometry varies according to acquisition parameters such as milliamperage, kilovoltage, and voxel size. As voxel size can be significantly small in X-ray microtomography, proper settings of those parameters become very important for the acquisition of higher-resolution, low-noise images [8].

Both quantitative and qualitative assessments of delicate structures such as root canals and the periodontal ligament require excellent imaging techniques [9]. For that purpose, one needs high-definition images that are usually acquired with small voxel sizes [10–12]. Voxel size is of paramount importance as it affects image quality, scanning time, and reconstruction time in cone-beam CT images. The influence of voxel size on image resolution is clearly defined in the literature, especially in diagnostic studies [13]. The voxel size in micro-CT scanners ranges from 5 to 50 *μ*m [7], and such small voxel sizes can generate images that are particularly valuable in the diagnosis of root fractures and root resorption [14]. Still, studies assessing root canal filling voids have arrived to very different conclusions mostly by the lack of standardization in terms of image resolution, voxel size, and other settings. The distance of axial scanning steps can be set according to the operator's intent and will affect both image resolution and exposure time. Indeed, while shortening of the scanning steps leads to longer X-ray exposure, it does produce images of higher resolution.

Therefore, the aim of this study was to determine a cutoff voxel size value for the assessment of root canal filling voids in micro-CT images as compared to those obtained with nano-CT scans as reference images.

## 2. Materials and Methods

The study protocol was in line with the Declaration of Helsinki, including all its amendments and revisions. The consent forms were reviewed and approved by the institutional board of research ethics and included permission to publish study subjects' photos and radiographs if necessary for scientific purposes. Study subjects or their proxies signed the consent forms prior to any intervention such as radiographic, intraoral, or extraoral examinations and tooth extractions. Freshly extracted mandibular central incisors were presorted with periapical radiographs to ensure they had a single, straight root canal. After that, the teeth were decoronated so that twenty 12 mm-long seperated roots were produced. The roots were then examined under an operatory microscope (OPMI pico; Zeiss Co., Jena, Germany) so as to select only those whose canal was round in shape.

A size number 10 K-File (Maillefer, Ballaiges, Switzerland) was inserted into the root canal until the tip was just visible through the apical foramen. The working length was determined by subtracting 0.5 mm from this length. The canals were instrumented using a crown-down technique with the EndoSequence rotary nickel titanium files (Brasseler USA, Savannah, GA), and the finishing file was number 40/0.06. Throughout the instrumentation steps, root canals were irrigated with 2 mL 5.25% NaOCl. The smear layer was then removed with 17% EDTA for 1 minute, followed by a final rinse with 3 mL of 5.25% NaOCl and 3 mL distilled water. The root canals were then dried up with paper points. A root canal sealer (AH Plus, Maillefer, Ballaiges, Switzerland) was prepared in accordance with the manufacturer's instructions and then filled according to the single-cone technique. Prior to scanning, the roots were stored at 37°C and in 100% humidity for 10 days to ensure the sealer was set.

### 2.1. Micro-CT Scanning

A high-resolution, desktop micro-CT system (Bruker Skyscan 1172, Kontich, Belgium) was used to scan the specimens. Settings were 100 kVp, 100 mA, and 0.5 mm Al/Cu filter; voxel sizes of 5.2, 8.1, 11.2, or 16.73 *μ*m; and 0.5 step rotation. To minimize ring artifacts, air calibration of the detector was carried out prior to each scanning. Each sample was rotated 360° within an integration time of 5 min. Mean scanning time was around two hours. Other settings included beam-hardening correction, as described, and input of optimal contrast limits according to the manufacturer's instructions and based on prior scanning and reconstruction of the specimens.

### 2.2. Nano-CT Scanning

Nano-CT scans were performed with the Phoenix NanoTom S system (GE Sensing & Inspection Technologies GmbH, Wunstorf, Germany) using tube voltage of 115 kV, current of 80 *μ*A, a 0.1 mm copper filter, and 500 ms of exposure at resolution of 1.5 or 5 *μ*m per voxel. Mean scanning time was around seven hours. Other settings included beam-hardening correction, as described, and input of optimal contrast limits according to the manufacturer's instructions and based on prior scanning and reconstruction of the specimens.

### 2.3. Imaging Analysis

NRecon (ver. 1.6.10.4, SkyScan, Kontich, Belgium) and CTAn (ver. 1.16.1.0, SkyScan, Aartselaar, Belgium) software were used to reconstruct and measure the samples as per the modified algorithm by Feldkamp et al. [15] to obtain two-dimensional (2D) axial images. For image reconstruction, ring artifact correction and smoothing were fixed to zero, and the beam hardening artifact correction was set to 40%. The scans were reconstructed to show 2D slices of the roots with the NRecon software (Skyscan, Kontich, Belgium). Several cross-sectional images were reconstructed from the whole volume in micro-CT (*n* = 1023) and nano-CT (*n* = 2400) scans. The CTAn software was used for 3D visualization and analysis of images acquired with micro-CT and nano-CT scanning.

The presence of voids was assessed in 2D slices according to the protocol suggested by Moeller et al. [16], whereby each section was evaluated on a 21.3-inch flat-panel, color-active matrix TFT medical display (NEC MultiSync MD215MG, Munich, Germany) with a resolution of 2048–2560 at 75 Hz and 0.17 mm dot pitch operated at 11.9 bits. An average of 254 cross-sectional images perpendicular to the long axis of the root were created, starting at the most apical part of the root at an interval of 0.5 mm. The resultant micro-CT images were then converted to TIFF files and coded. For nano-CT images, the interval was set to 0.5 mm, which resulted in an average of 596 cross-sectional images.

**Figure 1:**
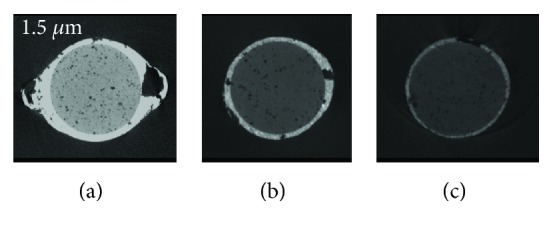
Root canal filling voids imaged with a 1.5 *μ*m voxel size in different regions of the root canal (a) apical, (b) middle, and (c) coronal thirds with nano-CT.

**Figure 2:**
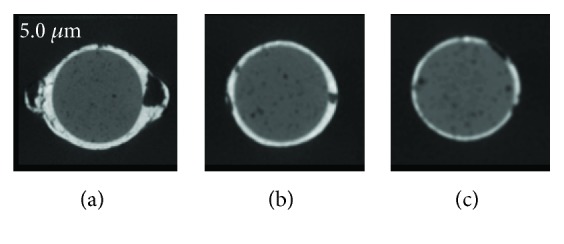
Root canal filling voids imaged with a 5.0 *μ*m voxel size in different regions of the root canal (a) apical, (b) middle, and (c) coronal thirds with nano-CT.

**Figure 3:**
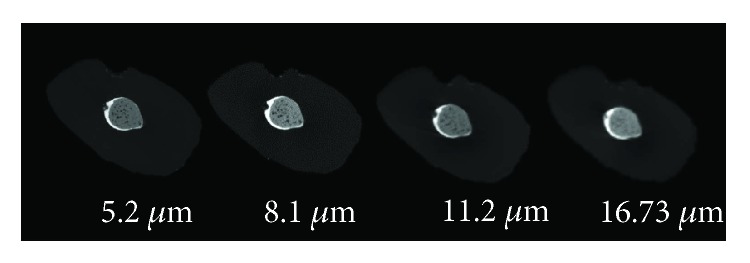
Root canal filling voids imaged with a 5.2/8.1/11.2 and 16.73 *μ*m with micro-CT.

Each section was assessed by three independent observers who used a binary registration scale: internal, external, and combined voids on nano-CT (Figures 1 and 2) and micro-CT images (Figure 3). Magnification was adjusted according to each observer's will. In case of disagreement, sections were reexamined until consensus was reached.

For volumetric calculation of the voids, the original greyscale images were processed with a Gaussian low-pass filter for noise reduction and an automatic segmentation threshold to subtract dentin from gutta-percha, sealer, and voids using the CTAn software. A thresholding (binarization) process was used, which entails processing the range of grey levels to obtain an image formed of black/white voxels only. Then, for each slice, a region of interest was chosen that contained a single object to allow for the volumetric calculation of the voids. For that calculation, each tooth was divided into thirds: apical (0 to 4 mm from the apical foramen), middle (4 to 8 mm from the apical foramen), and coronal (8 to 12 mm from the apical foramen).

Calculations included the percentage of root filling in terms of total volume, and the volume of voids inside the filling material (between gutta-percha and sealer), of those along the canal walls (between the sealer and canal wall), and of those voids that were a combination of the former two.

Differences among voxels were assessed with the Kruskal-Wallis test and the Mann–Whitney *U* test with the level of significance set to 5%.

## 3. Results

The results showed significant differences among the various voxel resolutions regarding the proportions of sections with voids and void volumes in micro-CT images (*p* < 0.05) but not in nano-CT ones (*p* > 0.05) (Table 1). Analysis of micro-CT images showed an increase in void formation in all root thirds, with a significant difference between 5.2/8.1/11.2, and 16.73 *μ*m voxel sizes (*p* < 0.05) (Table 2). Similarly, nano-CT voxel sizes did not produce different values for the chosen parameters from those obtained with micro-CT using voxel sizes of 5.2, 8.1, and 11.2 but did so for the voxel size of 16.73 *μ*m (*p* < 0.05) (Tables 1 and 2).

## 4. Discussion

Several conventional methods for assessing root canal filling voids have been presented in the literature, which include fluid filtration, dye penetration, radioisotopes, bacterial penetration, and saliva leakage. These studies have indicated that these conventional methods have disadvantages, such as being time-consuming and lacking standardization. For instance, the pressure used in the fluid filtration method cannot be properly controlled. Dye penetration studies, on the other hand, do not replicate the clinical situation faithfully and showed that air entrapped in the voids along the root canal filling may hinder fluid movement. Bacterial microleakage studies require long periods of observation and do not allow for the quantification of penetrating bacteria [17–19].

Given its higher accuracy and spatial resolution combined with its nondestructiveness, micro-CT analysis has been used in more recent studies for the evaluation of root canal filling quality and void presence. Moreover, this method allows for the distinction of gutta-percha, sealer, and tooth structure volumes at different levels of the dental roots (apical, middle, and coronal thirds) due to the possibility of using different greyscale levels [20]. Given these advantages, studies investigating endodontic filling quality using micro-CT analysis have become relatively frequent [21–24]. Still, selected segmentation threshold values may vary largely, and the objects of interest may vary along with the different settings used [25].

The feasibility of clinical CT studies on human teeth was initially suggested by Tachibana and Matsumoto [26]. Further development of CT studies was hampered mainly by low resolution of the images produced, which could not render proper reconstructions until significant improvements in software and hardware allowed for reduced slice thickness compared to those obtained with conventional CT ranges. Gambill et al. [27] and Rhodes et al. [28] used 1.5 *μ*m and 81 *μ*m slices with micro-CT systems, respectively. Dowker et al. [29] suggested that 5 *μ*m slices might be an attainable goal for in vitro investigations. Yet, Peters et al. [25] believed that a resolution of 34 and 68 *μ*m would be sufficient for endodontic micro-CT studies. Hence, studies using different voxel sizes for detecting root canal voids have delivered conflicting results. Indeed, resolution and voxel size are crucial to obtaining accurate results from thresholding and measurements, particularly in the calculation of endodontic filling voids. In this study, all teeth were scanned with different voxel sizes in the micro-CT (5.2, 8.1, 11.2, and 16.73 *μ*m) and in nano-CT (1.5 and 5.0 *μ*m) devices—the latter as the gold standard imaging modality—for the determination of a cutoff voxel value that allows for proper investigation of filling voids. The results showed significant differences among voxel resolutions regarding the proportions of sections with voids and void volumes in micro-CT images (*p* < 0.05), whereas no significant difference was found with the different voxel sizes in nano-CT images (*p* > 0.05). The analysis of micro-CT images showed an increase in void formation in all root thirds, with a significant difference after a cutoff value of 11.2 *μ*m (*p* < 0.05). As far as our review of the literature could go, there were no studies testing different voxel sizes for the assessment of endodontic filling voids. Jung et al. [20] conducted a study that assessed root canal fillings using micro-CT with 11 *μ*m resolution and concluded that the advantage of such a high resolution is greater accuracy of the rendered images. To obtain detailed information about delicate structures such as auxiliary gutta-percha cones, sealer thickness, or the presence of voids and lateral canals, high resolution seems to be beneficial. So far, this study was the first attempt to suggest a cutoff voxel size for imaging root canal filling voids, even though Jung et al. [20] stated that smaller voxel sizes result in higher resolution images, which is in line with this study. However, one should bear in mind that the higher the resolution, the more the data and the longer the scanning time.

Moreover, the quality of the 3D reconstruction of root fillings and sealers is determined by the spatial resolution and the grey-level spectrum of a given micro-CT system. It is difficult to confront micro-CT spatial resolution with sealer film thickness, because numerical data about sealer thickness vary considerably. Different filling techniques can produce sealer film thicknesses from 2.2 to 47.6 *μ*m. Thus, high-resolution three-dimensional scanning might be essential for that sort of investigation. Since filling techniques and root canal filling materials may also affect quantitative assessment of voids along with voxel sizes, further studies should be conducted in order to test these other variables.

## 5. Conclusions

In conclusion, all tested voxel sizes were capable to consistently detect root canal filling voids except for the 16.73 *μ*m voxel size in micro-CT. Within the limitation of this study, a voxel resolution of 11.2 *μ*m is suggested as the cutoff value in micro-CT and nano-CT imaging for the evaluation of root canal filling voids.

## Figures and Tables

**Table 1:** Mean percentage (and standard deviations) of section with voids, volume of root filling material, and percentage of internal, external, and combined voids in micro-CT and nano-CT images for each voxel size.

Voxel size (*μ*m)	Section with voids (%)	Filling material (%)	Internal voids (%)	External voids (%)	Combined voids (%)
1.5	79.4 (34.2)^a^	97.3 (1.3)^a^	0.73 (0.2)^a^	0.842 (0.6)^a^	0.983 (0.4)^a^
5.0	78.7 (38.5)^a^	97.6 (1.4)^a^	0.72 (0.2)^a^	0.731 (0.5)^a^	0.920 (0.5)^a^
5.2	78.3 (38.8)^a^	97.6 (1.3)^a^	0.70 (0.2)^a^	0.722 (0.5)^a^	0.928 (0.4)^a^
8.1	77.6 (34.7)^a^	97.7 (1.3)^a^	0.69 (0.2)^a^	0.702 (0.6)^a^	0.812 (0.5)^a^
11.2	73.2 (33.4)^a^	98.0 (1.3)^a^	0.67 (0.2)^a^	0.601 (0.6)^a^	0.693 (0.5)^a^
16.73	70.2 (36.5)^b^	98.8 (1.4)^b^	0.54 (0.2)^b^	0.325 (0.5)^b^	0.318 (0.5)^b^

Different letters indicate statistical difference between voxel size groups according to Kruskal-Wallis test and post hoc Mann–Whitney *U* test.

**Table 2:** Mean volumes (and standard deviations) of the root canal filling voids in different voxel sizes according to the apical, middle, and coronal thirds.

	Voxel size (*μ*m)	Apical	Middle	Cervical
Nano-CT	1.5	0.765 (0.3)^a^	0.825 (0.3)^a^	1.065 (0.5)^a^
	5.0	0.759 (0.3)^a^	0.774 (0.2)^a^	0.833 (0.3)^a^

Micro-CT	5.2	0.752 (0.3)^a^	0.77 (0.3)^a^	0.803 (0.5)^a^
	8.1	0.563 (0.2)^a^	0.744 (0.3)^a^	0.747 (0.4)^a^
	11.2	0.575 (0.2)^a^	0.694 (0.3)^a^	0.715 (0.5)^a^
	16.73	0.295 (0.4)^b^	0.353 (0.4)^b^	0.535 (0.9)^b^

Different letters indicate statistical difference between voxel size groups according to Kruskal-Wallis test and post hoc Mann–Whitney *U* test.

## Data Availability

The data used to support the findings of this study are available from the corresponding author upon request.
